# Ultrasound-guided percutaneous transhepatic cholecystic puncture and drainage for acute severe cholecystitis—a Case Report

**DOI:** 10.3389/fmed.2025.1585967

**Published:** 2025-07-03

**Authors:** Liqiang Li, Yunfei Zhang, Zihan Zeng, Liang Li, Jun Zhang

**Affiliations:** ^1^Department of General Surgery, The Second People’s Hospital of Hefei, Hefei Hospital Affiliated to Anhui Medical University, Hefei, China; ^2^Department of General Surgery, Hefei Second People’s Hospital Affiliated to Bengbu Medical University, Bengbu, China

**Keywords:** contraindications to surgery, cholecystocentesis and drainage, ultrasound guidance, acute severe cholecystitis, gallbladder stones

## Abstract

**Background:**

Patients with multiple underlying diseases, older patients, or those with temporary contraindications to surgery should not undergo laparoscopic cholecystectomy for the time being. Instead, percutaneous transhepatic cholecystic puncture and drainage with tube placement is the preferred option.

**Patient summary:**

A 55-year-old patient was admitted to the hospital due to “sudden upper abdominal pain and discomfort lasting for more than 1 day.” The issue began with epigastric pain and discomfort after consuming greasy food, leading to persistent bloating accompanied by nausea and vomiting of gastric contents. There were no chills or fever present. The patient initially sought treatment at the local county hospital, but symptomatic treatments there proved ineffective. Consequently, the patient and their family visited a local municipal tertiary hospital in an emergency. At the municipal hospital, it was recommended that the patient undergo a “laparoscopic cholecystectomy.” However, since the patient had undergone coronary stent implantation at the local county hospital on November 5, 2024, surgery was considered contraindicated. With acute severe cholecystitis symptoms worsening and pain relief efforts proving inadequate, the patient and family sought further treatment. After preoperative evaluations, the patient underwent a successful percutaneous transhepatic cholecystic puncture and drainage guided by color ultrasound. This procedure resulted in significant improvement in both abdominal pain and inflammatory markers.

**Conclusion:**

For patients undergoing surgical cardiac stent implantation, requiring prolonged anticoagulant therapy, and concurrently experiencing acute severe cholecystitis, percutaneous transhepatic gallbladder drainage (PTGD) is an effective treatment option.

## Introduction

Acute severe cholecystitis is a prevalent condition in general surgery (1), characterized by sudden onset and rapid progression. Calculous cholecystitis represents the most common form. Acute severe cholecystitis (ASC) often arises from inadequate and untimely intervention in cases of acute severe cholecystitis, leading to the spread of inflammation (2). Clinical evidence suggests that cholecystectomy is typically conducted on an emergency basis for patients with concomitant localized peritonitis. However, emergency surgical procedures often proceed without comprehensive auxiliary examinations and systematic preoperative evaluations. This lack of thorough preparation can lead to a higher incidence of intraoperative complications, such as bile leakage, hemorrhage, and the need for conversion to open surgery. These complications are often attributed to the presence of tissue inflammation and edema, which complicate the differentiation of anatomical structures (3, 4). For pregnant patients, those on anticoagulant medications, individuals who have recently undergone major surgeries such as cardiac surgery, and patients with acute severe cholecystitis who are temporarily unsuitable for LC, non-surgical management is commonly employed. This approach aims to alleviate symptoms, control infections, and prevent complications. Among these non-surgical treatments, PTGD is often preferred due to its effectiveness in managing the aforementioned conditions. Instead, clinical management should initially focus on reducing gallbladder pressure and alleviating the patient’s symptoms of infection (5–7). As society ages, the prevalence of elderly patients with multiple underlying comorbidities is steadily increasing, leading to heightened risks associated with surgical and anesthetic procedures. In recent years, the widespread advancement of ultrasound technology has facilitated the performance of percutaneous transhepatic gallbladder drainage (PTGD) under ultrasound guidance. This method effectively reduces gallbladder pressure and swiftly alleviates symptoms such as abdominal pain and fever. Therefore, for patients with ASC admitted on an emergency basis, immediate LC surgery is not recommended. The clinical priority should instead be to alleviate gallbladder pressure and address the patient’s infection symptoms promptly (8–11).

### Case presentation

Chief complaints: Sudden onset of epigastric pain and discomfort for more than 1 day.

History of present illness: A 55-year-old male presented with persistent upper abdominal distension and discomfort following the consumption of greasy food, experienced 1 day prior, accompanied by nausea and vomiting containing stomach contents. The patient reported no chills or fever, and denied palpitations or chest tightness. Initially, he sought symptomatic treatment at the local people’s hospital with negligible improvement. Consequently, he and his family sought emergency care at the municipal people’s hospital. An emergency abdominal CT scan revealed mild interstitial changes and large alveoli in both lungs, a gallbladder with increased volume and uneven density indicative of cholecystitis, fatty liver, and slightly dilated extrahepatic bile ducts. The emergency department recommended PTGD, which the patient and his family refused. Subsequently, seeking further treatment, he visited the emergency department of our hospital. Following evaluation, he was admitted with a diagnosis of “gallbladder stones with acute severe cholecystitis.” Throughout the course of his condition, the patient remained mentally alert, with no urinary or fecal incontinence and no significant weight change noted recently.

History of past illness: The patient was diagnosed with gallbladder stones5 years prior during a routine physical examination. One month ago, the patient sought medical attention at the local county people’s hospital due to sudden anterior chest pain. Following an electrocardiogram, cardiac stenting was recommended and subsequently performed. The patient was discharged post-operatively without complications. Personal and family history: There was no other relevant personal or family history.

Physical examination: The patient’s body temperature was 36.5°C; heart rate, 120 bpm; respiratory rate, 24 breaths/min; fingertip oxygen saturation (SpO_2_), 98%; and body weight, 90 kg. The skin and sclerae were free from jaundice, and there was no enlargement of the supraclavicular lymph nodes. Abdomen was distended, abdominal muscles were tense, right upper abdominal pressure (+), rebound pain (+), Murphy (+), liver and spleen were not palpated subcostally, gallbladder was not palpated subcostally, mobile turbidities (−), tenderness to percussion in hepatic region (−), bowel sounds were normal, 4 beats/min.

Laboratory examinations: Before the operation, the following biochemical tests were performed: total bilirubin, 60.6 μmol/L; alanine aminotransferase, 57.0 U/L; aspartate aminotransferase, 40.0 U/L; *γ*-glutamyltransferase, 109 U/L; White blood cell count:23.81^*^10^9^/L; Neutrophil percentage: 94.2%; Neutrophil absolute value: 22.43*10^9^/L.

### Imaging examinations

**Figure 1:**
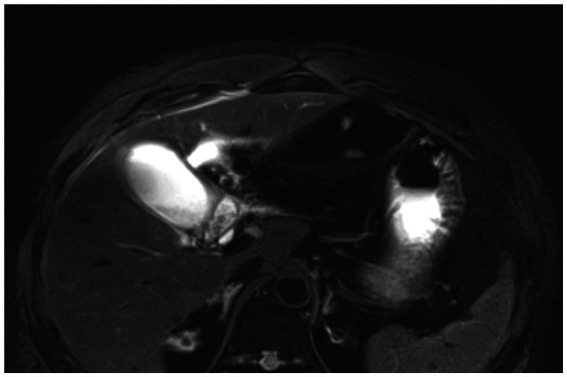
Abdominal MRCP results showed a full gallbladder volume with uneven density, manifestations of cholecystitis, fatty liver, and slightly dilated extrahepatic bile ducts.

A 55-year-old male patient was admitted to the hospital presenting with a sudden onset of epigastric pain and discomfort lasting more than 1 day. An emergency Magnetic Resonance Cholangiopancreatography (MRCP) examination of the abdomen was conducted, revealing the presence of multiple gallstones, gallbladder enlargement, and a minor accumulation of fluid (see Figure 1).

**Figure 2:**
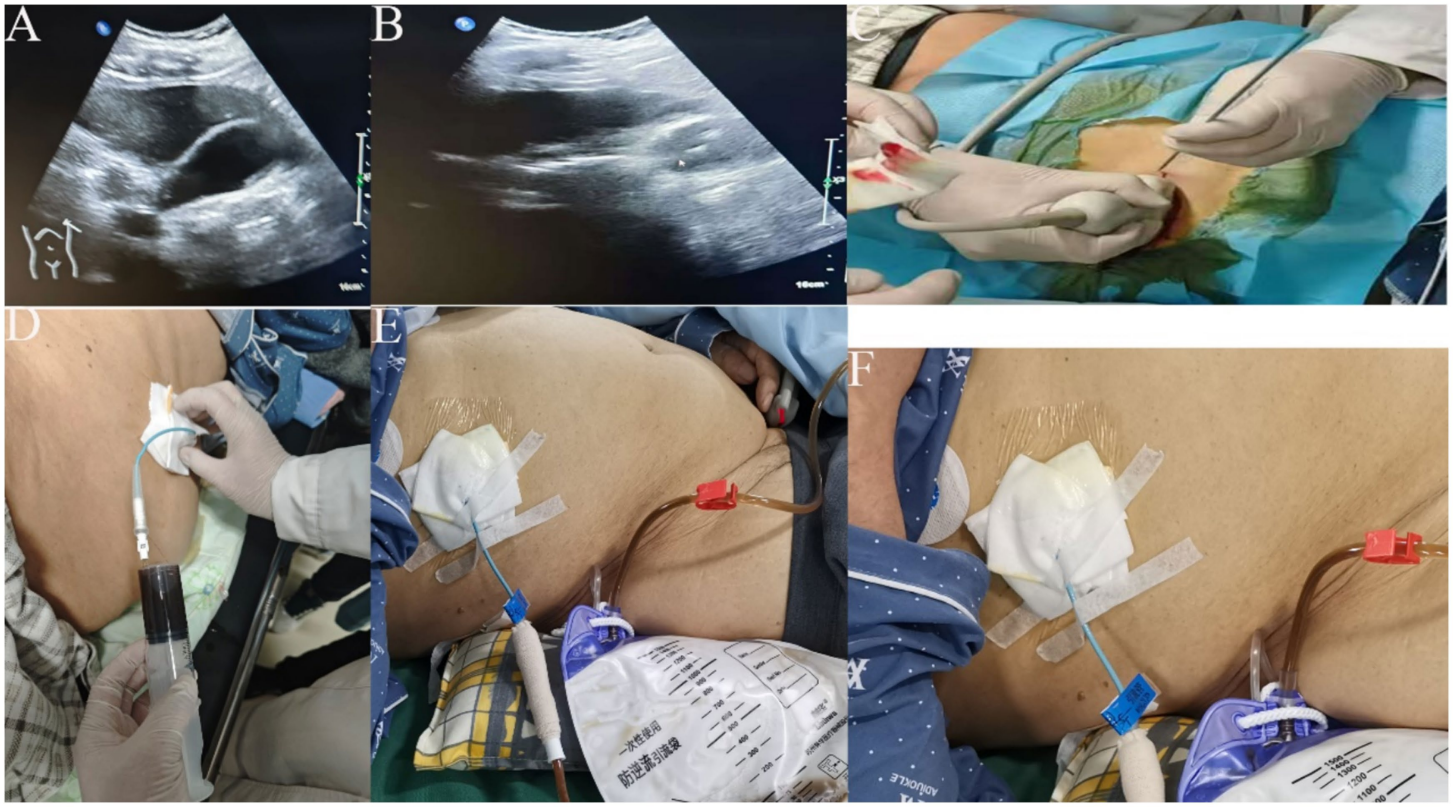
**(A,B)** ultrasound to clarify the location of gallbladder, liver, and stone; **(C)** ultrasound probe guided puncture needle through the liver to the gallbladder; **(D)** 20 mL syringe pumping back 8F to place the tube, see the bile can be stopped pumping back; **(E,F)** fix the guidewire and external drainage bag, record the flow of bile drainage every day.

1. This patient underwent routine blood, urine and fecal tests as well as biochemical tests. Routine fluid replacement was carried out to maintain acid-our hospital base balance. Patients were positioned either in a right lateral or supine position for the initial ultrasound examination (Figures 2A,B). The procedure was simulated, and the patient’s body was marked accordingly. After local disinfection and the application of sterile drapes, 5 mL of 5% lidocaine was used for local anesthesia. A 2–3 mm incision was made, and under ultrasound guidance, an 18-gauge puncture needle was gradually inserted into the gallbladder via the liver. A guide wire was placed, and following the dilation of the channel, an 8Fr pigtail catheter was positioned within the gallbladder. The guide wire was then withdrawn, and a drainage bag was attached (Figures 2C–F). Following percutaneous transhepatic gallbladder drainage (PTGD), the drainage tube was rinsed with metronidazole injection twice daily. The patient was monitored for potential complications, including pneumothorax and cholestatic peritonitis. The surgical procedure was smooth, with no adverse reactions experienced by the patient, who safely returned to the ward post-operation.

Postoperative precautions: ① Ensuring the protection of the drainage tube to prevent displacement; ② Assessing the feasibility of flushing if drainage is not smooth. ③Preventing postoperative infection and conducting 24-h cardiac monitoring.

Final diagnosis: 1. gallbladder stones with acute severe cholecystitis; 2. epigastric pain; 3. status after coronary stent implantation; 4. abdominal distension; 5. fatty liver; 6. dilatation of common bile duct.

Treatment: Patients with acute severe cholecystitis underwent percutaneous transhepatic gallbladder drainage (PTGD) surgery. Post-surgery, the patients demonstrated rapid recovery in biochemical indexes, reflecting significant clinical improvement. Moreover, symptoms such as abdominal pain and abdominal distension showed considerable alleviation. This procedure offered a palliative solution, paving the way for further treatment during follow-up.

### Outcome and follow-up

After the patient underwent PTGD surgery, an abdominal drainage tube was indwelled. The abdominal drainage tube drained approximately 200-300 mL of dark green bile every day, and no obvious abnormalities were observed in color and nature. Liver function and other tests were performed on the 5 day after surgery, and the following results were obtained: total bilirubin, 10.9 μmol/L; alanine aminotransferase, 44.5 U/L; aspartate aminotransferase, 20.2 U/L; *γ*-glutamyltransferase, 88.2 U/L; alkaline phosphatase, 56.5 U/L; White blood cell count:5.96*10^9^/L; Neutrophil percentage: 57.4%; Neutrophil absolute value: 3.43*10^9^/L. The patient’s liver function and color and characteristics of bile drainage from the drain were normal, leading to discharge on the 7 day. The patient will retain the PTGD tube (pigtail catheter) for an additional 2 months after discharge and will return to the hepatobiliary surgery department for tube removal at the end of this period. During the tube retention period, the patient took care to protect the PTGD tube, and no complications, such as infection, or dislodgement, were observed. The PTGD tube was removed and the patient continued to control his diet; because the patient had undergone “coronary stenting” 3 months ago and was taking oral anticoagulant medication at the same time, it was not advisable to perform minimally invasive surgery within 6 months, and the patient returned to the hospital for minimally invasive surgery after 6 months (12).

## Discussion

The prognosis for acute severe cholecystitis is significantly influenced by the treatment method employed during the acute phase. For patients with acute severe cholecystitis, especially those who have been taking anticoagulant drugs for a long time and have recently undergone major surgical operations, PTGD is the best choice (13–15). For patients who have undergone coronary artery stent implantation and require lifelong anticoagulation therapy, as well as those who need to continue specific medications, there is a significant challenge. They often must suspend these medications for more than 6 days before undergoing any surgical treatment (16); lower common bile duct obstruction with enlarged gallbladder, which cannot be treated surgically or fails to be drained by biliary drainage (17); pregnancy; acute severe cholecystitis and enlarged gallbladder with the risk of perforation (18, 19).

a patient underwent major cardiac surgery and requires long-term administration of anticoagulant medications. At this time, the high adhesion of the gallbladder triangle with ASC in the patient makes it difficult to disanatomy during the operation and is very likely to damage the blood vessels, which can lead to an increase in the rate of conversion to laparotomy and the incidence of common bile duct injury. Therefore, for patients who have been taking anticoagulant drugs for a long time, LC remains challenging. However, PTGD can achieve gallbladder decompression through external drainage, relieve the tension of the gallbladder, reduce toxin absorption, and improve inflammatory symptoms. It can be completed under the guidance of B-ultrasound. At the same time, combined with antibiotic treatment can quickly relieve the symptoms and signs of patients and prevent the deterioration of the condition. Create favorable conditions for the elective LC (20).

According to the disease-specific severity classification provided by the Tokyo Guidelines 2018, the patient fulfilled the criteria for Grade II (moderate) acute severe cholecystitis. Based on these guidelines, patients presenting with this condition are candidates for either emergency LC or PTGD. However, given the patient’s recent cardiac stent placement 3 months prior, LC is contraindicated at this time. Due to its characteristics such as simple operation, small trauma, low complication rate and low mortality rate, PTGD has been confirmed by multiple clinical applications at home and abroad as a low-risk treatment method for acute severe acute severe cholecystitis, and it has been affirmed that this drainage surgery creates favorable conditions for secondary cholecystectomy (7, 21, 22).

In the context of ASC, various inflammatory cells, including monocytes, macrophages, lymphocytes, and neutrophils, generate substantial quantities of cytokines. In this case, the patient exhibited significantly elevated white blood cell and neutrophil counts before intervention. Following PTGD, these inflammatory indicators were effectively managed, with the patient’s pain score reduced from 6 to 4 post-procedure.

Previous studies have demonstrated that for patients with Grade II AC, performing a delayed LC following initial PTGD considerably reduces the rates of conversion to open surgery and postoperative complications—such as bile duct injury, bile leakage, intra-abdominal fluid accumulation—compared to emergency LC, with statistically significant differences observed (10). Consequently, undertaking LC subsequent to PTGD not only diminishes surgical complications but also alleviates patient discomfort, contributing to improved overall outcomes.

However, PTGD is not without drawbacks; common complications include bile peritonitis, pneumothorax, hemorrhage, gastrointestinal injury, and dislodgement of the drainage tube. Bile leakage poses a particularly serious risk during PTGD puncture. Such complications can induce significant psychological and mental distress in patients and may necessitate emergency LC, thereby increasing the patient’s financial burden.

Postoperatively, patients live with a PTGD tube, which can substantially impact their daily living. Inadequate care of the PTGD tube post-surgery can significantly elevate the risk of biliary infections, with the potential for tube dislodgement leading to biliary peritonitis. In this specific case, the patient was required to maintain the PTGD tube for up to 4 to 6 weeks or even longer inadvertently increasing both financial and psychological stresses. This scenario highlights one of the inherent disadvantages of prolonged PTGD tube use.

Ultrasound-guided PTGD is considered the treatment of choice for high-risk patients with acute severe cholecystitis. This method has demonstrated exceptional efficacy in alleviating biliary infections, with reported success rates ranging from 98.6 to 100.0%. The associated risks include puncture and drainage complications at a rate of 4.1%, and the mortality rate is notably low, at only 1.4% (7, 23). Previous studies have found that 32 to 53% of patients were febrile at the time of presentation and 51 to 53% presented with leukocytosis (24, 25). In this case report, our findings indicate that leukocyte counts generally decreased in patients undergoing PTGD.

In recent years, ultrasound-guided PTGD has been extensively utilized as a method of intracystic drainage, particularly benefiting patients of Long-term use of anticoagulant drugs and those with numerous underlying conditions who are unable to undergo cholecystectomy. This case report highlights the advantages of ultrasound-guided PTGD, which include reduced procedure duration, expedited extubation, rapid infection control.

## Conclusion

This case report illustrates that PTGD is a safe and effective treatment for patients with acute moderate or severe cholecystitis. Through this case, we can find that PTGD is the best option for patients with Grade II or above, those who have recently undergone major surgeries, and those who have been taking anticoagulant drugs for a long time and are temporarily unable to undergo LC surgery.

## Data Availability

The original contributions presented in the study are included in the article/supplementary material, further inquiries can be directed to the corresponding author.
